# Transperineal Laser Ablation for Benign Prostatic Enlargement: A Systematic Review and Pooled Analysis of Pilot Studies

**DOI:** 10.3390/jcm12051860

**Published:** 2023-02-26

**Authors:** Alessandro Tafuri, Andrea Panunzio, Francesco De Carlo, Elia Luperto, Federica Di Cosmo, Arturo Cavaliere, Mino Rizzo, Zhe Tian, Aliasger Shakir, Rita De Mitri, Antonio Benito Porcaro, Maria Angela Cerruto, Alessandro Antonelli, Luigi Cormio, Giuseppe Carrieri, Pierre I. Karakiewicz, Andre Luis Abreu, Vincenzo Pagliarulo

**Affiliations:** 1 Department of Urology, “Vito Fazzi” Hospital, 73100 Lecce, Italy; 2 Department of Urology, Azienda Ospedaliera Universitaria Integrata Verona, University of Verona, 37126 Verona, Italy; 3 Cancer Prognostics and Health Outcomes Unit, Division of Urology, University of Montréal Health Center, Montréal, QC H2X 0A9, Canada; 4 Catherine and Joseph Aresty Department of Urology, USC Institute of Urology, Keck School of Medicine, University of Southern California, Los Angeles, CA 90033, USA; 5 Department of Urology and Renal Transplantation, University of Foggia, 71122 Foggia, Italy

**Keywords:** prostatic diseases, minimally invasive surgical procedures, laser ablation, outcomes assessment

## Abstract

Transperineal laser ablation (TPLA) of the prostate is a new minimally invasive treatment option in men with lower urinary tract symptoms (LUTS) due to benign prostatic enlargement (BPE). The aim of this systematic review was to investigate the efficacy and safety of TPLA in the management of BPE. The primary outcomes were the improvement in urodynamic parameters (maximum urinary flow (Q_max_) and postvoiding residue (PVR)) and LUTS relief, assessed using the IPSS questionnaire. The secondary outcomes were the preservation of sexual and ejaculatory functions, assessed with the IEEF-5 and MSHQ-EjD questionnaires, respectively, and rates of postoperative complications. We reviewed the literature for prospective or retrospective studies evaluating the use of TPLA in the treatment of BPE. A comprehensive search in PubMed, Scopus, Web of Science, and ClinicalTrials.gov was performed for English language articles published between January 2000 and June 2022. Pooled analysis of the included studies with available follow-up data for the outcomes of interest was additionally performed. After screening 49 records, six full-text manuscripts were identified, including two retrospective and four prospective non-comparative studies. Overall, 297 patients were included. All the studies independently reported a statistically significant improvement, from baseline, in Q_max_, PVR, and IPSS score at each timepoint. Three studies additionally demonstrated that TPLA did not affect sexual function, reporting no change in the IEEF-5 score, and a statistically significant improvement in MSHQ-EjD score at each timepoint. Low rates of complications were recorded in all the included studies. Pooled analysis showed a clinically meaningful improvement in both micturition and sexual outcomes mean values at 1, 3, 6, and 12 months of follow-up, compared with baseline. Transperineal laser ablation of the prostate for the treatment of BPE showed interesting results in pilot studies. However, higher level and comparative studies are needed to confirm its efficacy in relieving obstructive symptoms and preserving sexual function.

## 1. Introduction

Lower urinary tract symptoms (LUTS) are a frequent cause of inconvenience, impair quality of life, and are often associated with bladder outlet obstruction related to benign prostatic enlargement (BPE) [1]. The natural history of BPE is progressive, and if untreated, it may cause major complications such as acute urinary retention, hydronephrosis, and acute kidney injury. Medical therapy, including alpha-1-adrenoceptor antagonists, 5-alpha-reductase inhibitors, muscarinic receptor antagonists, beta-3 agonists, and phosphodiesterase-5 inhibitors, as well as plant extracts, is recommended by international guidelines and currently represents the first line in the management algorithm of male LUTS [1]. In case of intolerance, poor compliance, or lack of efficacy of medical therapy, several surgical treatment options are available, in order to remove prostatic obstruction and to improve patient quality of life. Advancements in endoscopic technology have allowed the development of an increasing number of new approaches for the treatment of BPE, such as monopolar and bipolar transurethral resection of the prostate (TURP). The most important improvements have been reported in endoscopic laser treatments of BPE, and prostate laser enucleation through different energy sources has been widely studied and has proved effective but not without adverse side effects or sequelae [2,3,4]. Importantly, these treatments are often related to a high rate of anejaculation or retrograde ejaculation, which is not negligible in sexually active patients [5]. Minimally invasive treatment options, including prostatic artery embolization, UroLift, temporary implantable nitinol device, Rezum, and intraprostatic injection, have also showed fast and effective relief of LUTS without affecting quality of life in carefully selected patients [6]. Although these procedures achieve inferior improvements in functional outcomes compared with standard transurethral treatments, they have the advantage of being performed in the office using local anesthesia or intravenous or oral sedation [6].

In the last few years, transperineal laser ablation (TPLA) of the prostate has been proposed as a new minimally invasive treatment option for BPE, and it is currently under investigation in order to evaluate urodynamic improvements and patient symptom relief [7,8,9,10,11,12]. Here, we performed a systematic review and pooled analysis of studies that have reported data on TPLA for BPE.

## 2. Materials and Methods

### 2.1. Search Strategy

This systematic review was performed following the Preferred Reporting Items for Systematic Review and Meta-Analyses (PRISMA) statement [13]. A comprehensive search in PubMed, Scopus, Web of Science, and ClinicalTrials.gov was performed for English language articles published between January 2000 and June 2022 focused on TPLA for BPE. The key terms used for the search were as follows: ((benign prostatic obstruction) OR (BPO) OR (benign prostatic hyperplasia) OR (BPH) OR (benign prostatic enlargement) OR (BPE) OR (lower urinary tract symptoms) OR (LUTS)) AND (transperineal laser treatment). An additional search using Google Scholar was performed to identify supplementary studies of interest not yet included in the other databases. The present study was registered with PROSPERO (International Prospective Register of Systematic Reviews) under the registration code CRD42022336253.

### 2.2. Selection of Eligible Studies, Quality of Studies and Risk of Bias Assessment, and Data Extraction

Two paired investigators (A.P. and A.T.) independently screened all title and abstract records gathered from the literature review to identify potential eligible studies, and then evaluated full-text manuscripts to determine the final included ones. Any disagreements about eligibility were resolved by discussion between the two investigators until a consensus was reached. We selected only prospective studies or retrospective evaluations, including more than 20 patients, and reporting the outcomes of interest. Non-English articles, editorial commentaries, articles focused on other drugs or diseases, and clinical trials with no provided publication were excluded. The PICOTS (Population, Intervention, Comparators, Outcomes, Timing, and Setting) format [14] was used to scrupulously summarize our research and analysis strategy for evaluating the outcomes of interest (Supplementary Table S1). 

All the articles were categorized according to level of evidence using both the Oxford Level of Evidence Working Group 2011 [15] and the GRADE (Grading of Recommendations Assessment, Development, and Evaluation) systems [16]. The quality of the studies was assessed using the Newcastle-Ottawa scale for non-randomized studies (total score ≤5: low quality; 6–7: intermediate quality; 8–9: high quality) [17] (Supplementary Table S2).

Risk of bias was independently assessed by two paired investigators (A.P. and A.T.) for all the included studies using Cochrane tools for non-randomized studies [18]. Risk of bias assessment was then generated with the ROBINS-I tool [19] (Supplementary Figure S1).

All data extracted from the included studies were recorded in an electronic database. Collected data included main author and year of publication, country of origin, type of laser used, number and age of enrolled patients, prior therapies received, and outcomes measured. The primary outcomes were the improvement in urodynamic parameters (maximum urinary flow (Q_max_) and postvoiding residue (PVR)) and symptom relief, assessed using the International Prostatic Symptoms Score (IPPS) questionnaire [20], comparing follow-up data with baseline patient characteristics. The secondary outcomes were the preservation of sexual function considering both erection and ejaculation, and rates of intraoperative and postoperative complications. 

### 2.3. Statistical Analyses

For continuous coded variables reported as median, results were converted to mean ± standard deviation (SD). After obtaining the mean ± SD, data were converted to mean with 95% confidence interval (CI). A pooled analysis of the means (95% CI) was performed for the studies that reported the outcome of interest at a specified timepoint. The random effects model was used to evaluate the I^2^ value for heterogeneity. The R software environment for statistical computing and graphics (R version 4.1.2, R Foundation for Statistical Computing, Vienna, Austria) was used for all analyses.

## 3. Results

**Figure 1:**
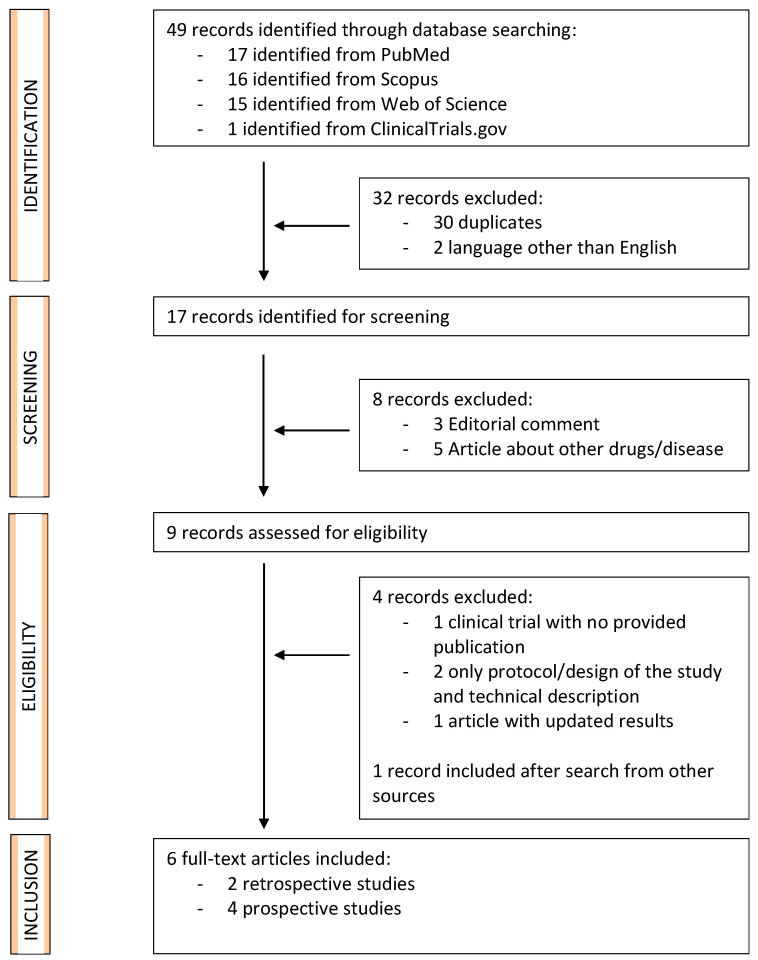
PRISMA (Preferred Reporting Items for Systematic Review and Meta-Analyses) flow diagram for identification and selection of studies assessing the efficacy of transperineal laser ablation (TPLA) for benign prostatic enlargement (BPE).

The PRISMA diagram shows the literature research results (Figure 1). We identified 49 records overall for screening. A total of nine records were retrieved and assessed for their eligibility. One study that did not provide clinical results (NCT03653117) and two studies that only focused on description of the technical aspects of TPLA were excluded [21,22]. Also, the preliminary report by Patelli et al. [23] was subsequently updated by Pacella et al. [7], and only the latest version was included. Finally, six full-text manuscripts, including four prospective studies, one retrospective single-center study, and one retrospective multi-institution study, met the inclusion criteria and were included [7,8,9,10,11,12]. Data on 297 patients treated with TPLA due to BPE were reported among included studies.

### 3.1. Patient Selection

Recognizing the right patient setting for the prostate TPLA approach has a pivotal role in reaching functional outcomes (Table 1). Five studies specified the inclusion and exclusion criteria to be adopted for TPLA. The only exception was Sessa et al. [12]. Inclusion criteria consisted of age > 50 years in three studies [7,9,10], between 40 and 90 years in one study [8], and ≥45 years in one study [11]. In all the studies, the IPSS questionnaire was used to evaluate the severity of LUTS and quality of life (QoL). Specifically, a cut-off score of 12 was adopted in four studies [7,8,9,10], and a cut-off score of 8 was adopted in the other one [11]. A general agreement on prostate volume >30 mL, using magnetic resonance imaging (MRI) or ultrasonography (US), was achieved. Urodynamics parameter values, such as a Q_max_ ≤ 15 mL/s and a PVR ranging between 50 mL and 400 mL, were used. In addition, lack of efficacy or intolerance to previous medical therapy was reported in two studies [8,10], and a prostate-specific antigen (PSA) value < 4 ng/mL, a previous negative prostate biopsy, or a negative digital rectal examination (DRE) were reported in one study [11]. Patients with a history of previous prostate surgery, indwelling catheter or intermittent catheterization, presence of bladder stones, detrusor acontractility or hypocontractility, urethral strictures, neurogenic bladder dysfunctions, previous diagnosis of bladder cancer or prostate cancer, PSA ≥ 4 ng/mL, or clinical or imaging findings suspicious for malignancy confirmed by biopsy were excluded from all the studies. Additionally, Manenti et al. reported the presence of a large median lobe as an exclusion criterion [10]. Conversely, Cai et al. considered, among exclusion criteria, hypersensitivity to US contrast media [9].

### 3.2. Technical Aspects of TPLA

In all the included studies, TPLA was performed using EchoLaser^TM^ (SoracteLite^TM^). Technical equipment included a diode-laser generator device, Echolaser XVG system (EchoLaser X4 in addition to EchoLaser smart interface; Elesta s.r.l., Calenzano (FI), Italy), and a biplanar transrectal ultrasound (TRUS) probe.

Depending on the prostate size, up to two 21 Gouge Chiba needles for each lobe were introduced transperineally, under US guidance, allowing the subsequent positioning of 300 or 272 μm bare flat-tip optical laser fibers (Table 2). The optical fibers were then connected with a continuous wave diode laser source, operating at a 1064 nm wavelength, with four independent devices for firing the prostatic tissue simultaneously. The introducer needle was designed to expose the fiber tip of 5 mm. Applicators were positioned along a path that was as parallel as possible to the longitudinal plane of the prostate, according to the relation of the urethral position and its longitudinal width, to generate a symmetric cavity of ablation, to reduce urethral stromal compression, and to shift the urethral lumen as close as possible to the midline. Positions were always confirmed in real time, and eventually modified using the biplanar US device. 

In all the studies, a standard number of one needle per lobe was used; additional needles were placed if the prostate volume was ≥80 mL [12], ≥60 mL [11], ≥45 mL [10], or ≥40 mL [7]. De Rienzo et al. proposed one more needle per lobe if the prostate volume was ≥55 mL, and one more needle if a median lobe was also present [8]. As a rule, needle positioning had to consider a distance not inferior to 8 mm from the urethra lumen and from the prostatic capsule, and a distance greater than 15 mm from the bladder neck. Additionally, 10–15 mm between two needles had to be maintained, when more than one needle was positioned in the same lobe. In order to ease the insertion of the needles, De Rienzo et al. used a transrectal US biplanar probe combined with a multichannel needle applicator, with a dedicated software displaying a grid overlaying the US image [8]. 

In three of the six studies [7,9,11], each treatment session was performed using a fixed power of 3 or 3.5 Watt (W). Conversely, Manenti et al. reported a fixed power protocol of 3 W after an initial 2-min 5 W pulse ablation [10]. Similarly, De Rienzo et al. adopted a starting power of 4.5 W, then reduced to 3.5 W after 1–2 min, when bubbles of vaporized tissue became visible at US [8]. Finally, Sessa et al. relied on a starting power of 5 W reduced to 3.5 W after 2 min [12]. Overall, ablation time ranged from a minimum of 400 s to a maximum of 600 s, to maintain the total energy applied between 1200 and 1800 Joule per fiber. Depending on the size of the prostate, one to two consecutive illumination cycles were performed during the same treatment session. During the procedure, energy delivery parameters were monitored by the operator through a display on the laser machine, and the progress of ablation was monitored by US. Treatment was concluded when the gas forming during the ablation had covered the entire desired area and appeared as a hypoechogenic US image (‘‘pull-back’’ technique), or when 1800 Joule per illuminations was reached. After the ablation, Cai et al. proposed the evaluation of the ablation area by contrast-enhanced US [9]. 

### 3.3. Management of Perioperative Patients

The rate of patients treated preoperatively with medical therapy is reported in Table 3. Before the procedure, all patients underwent a routine blood exam, including standard coagulation tests. In the study of Manenti et al., all patients underwent MRI preoperatively to measure the prostate volume and to assess the morphological characteristics of BPE, as well as one hour after TPLA to evaluate the extension of the treated zone. 

In all the studies, patients were placed in lithotomy position. A three-way catheter was always placed, allowing the continuous saline irrigation of the urethra and bladder during the entire procedure. In four of the six studies (233 patients) [7,8,11,12], TPLA was performed under conscious sedation (midazolam 3 or 4 mg), in addition to perineal and periprostatic anesthesia (20 mL lidocaine solution 2%). In the other two studies (64 patients), local anesthesia only was used [9,10]. Sessa et al. also recommended the application of an anesthetic cream on the perineum skin [12] (Table 3).

In five studies [7,8,10,11,12], antibiotic prophylaxis was administered one hour or the day before the treatment and was then continued for the subsequent 5–7 days. Cephalosporines (cephazolin 2 g/cefixime 400 mg) or fluoroquinolones (ciprofloxacin 500 mg/levofloxacin 500 mg) were usually used. No data regarding type, dose, and time of antibiotic administration were reported by Cai et al. [9]. In three studies [10,11,12], a single dose of dexamethasone 8 mg or methylprednisolone 20 mg was intraoperatively administered to reduce postprocedural prostatic edema. 

Operative time ranged from 28.2 to 60.9 min. Cai et al. and Frego et al. specifically reported an ablation time of 42.6 min and 17.2 min (1033 s), respectively [9,11]. Length of hospital stay (LOS) varied according to studies. In the studies of Manenti et al. and Sessa et al., patients were discharged 2–3 h after the treatment [10,12]. Conversely, in the other studies patients were kept in hospital for 1–2 days. Median LOS ranged from 6.4 h [12] to 1.8 days [7]. Corticosteroid therapy (prednisone 25 mg) was continued for 5–15 days with the progressive tapering of the dose, according to studies [8,10,11]. Finally, both Manenti et al. and De Rienzo et al. recommended the continuation of the alpha-blocker therapy until the 30th postoperative day [8,10]. In all such studies, patients were discharged with an indwelling catheter, and its removal was recommended after seven days. Catheterization time ranged from 7 to 16.5 days [7,8,9,11,12].

### 3.4. Functional Outcomes

Preoperative data on Q_max_, PVR assessed by transabdominal US, prostate volume assessed by TRUS or MRI, and IPSS and QoL scores were available for all patients. Additionally, IIEF-5 (International Index of Erectile Function [24]) and MSHQ-EjD (Male Sexual Health Questionnaire-Ejaculatory Dysfunction [25]) scores were reported in four [8,9,11,12] and three studies [8,10,12], respectively. The follow-up schedule usually consisted of a visit, US or MRI evaluation, and the patient filling out all the dedicated questionnaires. According to studies, the outcomes of interest were reported at baseline, and after 1, 3, 6, or 12 months of follow-up, as shown in Table 4.

All studies independently demonstrated a statistically significant improvement from baseline values in mean or median Q_max_ and PVR at each specified timepoint (Table 4). Only in Frego et al.’s study, PVR improvement during the follow-up failed to reach statistical significance, although a clinically meaningful reduction was proved at 3, 6, and 12 months [11]. Similarly, a statistically significant improvement from baseline values was recorded for IPSS and QoL scores in all the included studies. Specifically, the highest reduction in IPPS score was observed by Frego et al. (Δ = −16.0 at 12 months) [11], while the highest decrease in QoL score was observed by Manenti et al. (Δ = −3.7 at 12 months) [10].

Four studies provided complete follow-up data on erectile function [8,10,11,12]. Specifically, no change in IIEF-5 score was shown at each specified timepoint in all the reports. Finally, three studies [8,10,12] also evaluated the ejaculatory function using the MSHQ-EjD. In all these studies, ejaculatory function was not only preserved but also improved, as showed by a statistically significant increase in MSHQ-EjD score at each specified timepoint (Δ = +3.9 at 3 months, Δ = +2.9 at 6 months, and Δ = +2.8 at 12 months, in Sessa et al., De Rienzo et al., and Manenti et al., respectively) [8,10,12].

### 3.5. Complications

Among the included studies, only Cai et al. [9] reported the occurrence of an intraoperative complication consisting of urethral burn that was treated by keeping the bladder catheter for 25 days. All the studies reported type and number of postoperative complications according to the Clavien–Dindo classification system [26] (Supplementary Table S3). De Rienzo et al. experienced a case (4.8%) of prostatic abscess treated with percutaneous drainage and antibiotic therapy [8]. Manenti et al. had three (6.8%) postoperative complications: a case of hematuria managed by keeping the bladder catheter for seven days, a case of orchitis treated with antibiotic therapy, and a case of prostatic abscess treated with percutaneous drainage and antibiotic therapy [10]. Pacella et al. reported eight (4.9%) cases of postoperative complications: three cases of hematuria managed with the bladder catheter being left in place for 15 days, three cases of acute urinary retention, a case of orchitis treated with antibiotic therapy, and a case of prostatic abscess, which was successfully drained [7]. Finally, 6 patients (3.7%) experienced transient dysuria, and 2 patients (1.2%) independently reported loss of ejaculatory function at follow-up visits. Dysuria and ejaculatory disorders were regarded as sequelae [7]. No detailed description of postoperative complications or sequelae was provided by Sessa et al.; however, these authors specified that no Clavien–Dindo ≥2 complications occurred [12]. 

### 3.6. Pooled Analysis

#### 3.6.1. Micturition Outcomes

**Figure 2:**
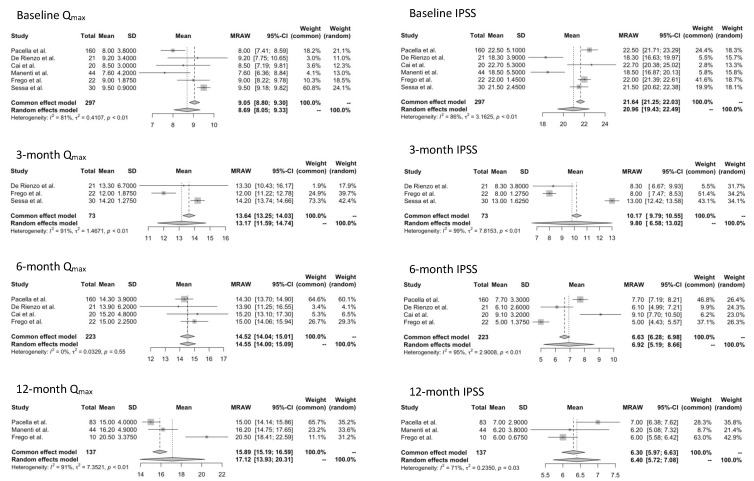
Forest plots illustrating the pooled mean and 95% confidence interval (CI) for maximum flow rate (Q_max_) and International Prostatic Symptoms Score (IPSS) at baseline and during follow-up [7,8,9,10,11,12].

At baseline, the overall pooled mean for Q_max_ was 8.69 mL/s, and an improvement was recorded during the follow-up at 3 (13.17 mL/s), 6 (14.55 mL/s), and 12 months (17.12 mL/s) (Figure 2). Pooled mean for PVR decreased from 91.94 mL at baseline to 36.0 mL at 3 months, to 27.57 mL at 6 months, and to 22.27 mL at 12 months (Supplementary Figure S2). A clinically meaningful improvement in the overall pooled mean for IPPS score from 20.96 at baseline to 9.80 at 3 months was observed and remained relatively stable even at 6 months (6.92) and 12 months (6.40) (Figure 2). Similarly, the overall pooled mean for QoL score decreased from 4.52 at baseline to 1.47 at 3 months and remained relatively stable even at 6 months (1.66) and 12 months (1.55) (Supplementary Figure S2).

#### 3.6.2. Sexual Outcomes

Data for IIEF-5 and MSHQ-EjD were available only in three studies. The overall pooled mean value for IEEF-5 score at baseline of 18.35 remained stable during the follow-up at 1 month (17.98) and 3 months (20.54). The pooled mean for MSHQ-EjD increased from 5.08 at baseline to 7.34 at 1 month and 7.95 at 3 months (Supplementary Figure S3).

## 4. Discussion

Based on the main data from the examined studies, the optimal candidates for TPLA of the prostate are patients aged ≥ 50, with a prostate volume ranging between 30 and 100 mL, and moderate to severe LUTS due to BPE, defined as the presence of an IPSS > 12, a Q_max_ < 15 mL/min, and a PVR > 30 mL, or refractory to previous medical therapies. All patients should start the antibiotic prophylaxis one day before the procedure and continue for seven days after. Similarly, the intraoperative administration of corticosteroid therapy to be continued for 7–15 days after the procedure should be recommended to avoid irritative symptoms. Patients may be discharged the day after the treatment with the bladder catheter, to be removed after one week in the absence of complications. The first follow-up visit may be scheduled within three months of the procedure and should consist in performing uroflowmetry and US and filling out the IPPS questionnaire.

In all the included pilot studies, a statistically significant improvement from baseline was independently observed for Q_max_, IPSS, and PVR at each specified timepoint. Similarly, when data were aggregated in pooled analysis according to specific timepoints for the available studies, a clinically meaningful improvement was also recorded for each of these micturition outcomes. These findings are in agreement with previous results reported by Checcucci et al. regarding ultra-minimally invasive surgical treatments of BPE [6]. Additionally, all six studies showed a statistically significant QoL improvement after TPLA at all specified timepoints. De Rienzo et al. evaluated the functional outcomes after 1, 3, and 6 months of follow-up, showing a statistically significant improvement at 1 month but even more evident at 3 and 6 months. These authors supposed that progressive improvement can be explained by the inflammatory effect of lasing and coagulative necrosis, which can partially hinder the beneficial effects immediately after the procedure [8]. 

Interestingly, considering sexual outcomes, it has been demonstrated that TPLA did not affect erectile function according to IIEF-5 score values. Additionally, De Rienzo et al. found a statistically significant improvement in MSHQ-EjD3 score at 1, 3, and 6 months. Authors also recorded ejaculatory discomfort at 1 month, no longer observed at successive follow-up, probably as a consequence of the inflammatory response that was treated using anti-inflammatory drugs [8]. Similarly, Sessa et al. and Manenti et al. found a significant MSHQ-EjD3 improvement at 1, 3, and 12 months, respectively [10,12]. Pooled analysis showed an increase in MSHQ-EjD3 score from baseline at 1 month and 3 months. These results are innovative and demonstrate a possible superiority of TPLA with respect to other standard techniques for BPE treatment [5]. The sexual/ejaculatory function improvement might be related to bladder neck preservation [27], as well as to the preservation of the muscular tissue around the verumontanum and particularly its proximal part implicated in the contraction of the external sphincter coordinated with the bulbar urethra [27]. Additionally, the reduction of urethra compression after treatment causes an ejaculatory flow-strength improvement. In this context, Manenti et al. showed an MRI-detected prostate volume reduction of more than 50% at 12 months after TPLA [10].

Finally, all the included studies showed that TPLA is a feasible and safe technique for BPE treatment related to short operative time duration, short LOS, and low intraoperative and postoperative rate of complications, thus allowing this procedure to be performed in an outpatient setting [6]. 

Taken together, preliminary data from these pilot studies suggest that TPLA represents a valid option for BPE due to its effects on LUTS relief and benign prostatic obstruction removal, the possibility of minimizing the need for anesthesia in the operating room, the short length of hospitalization, and the low rates of high-grade complications. However, the clinical improvements achieved with this technique are lower than those reported by standard treatments, and long-term data on TPLA efficacy as well as data on surgical reintervention rates are missing; furthermore, no conclusion can be achieved regarding the durability of the effect of this technique. On the other hand, the preservation of ejaculatory function is independently reported by all the included studies using the MSHQ-EjD questionnaire. TPLA might be considered for people interested in preserving sexual and ejaculatory functions, although its preoperative standard assessment that also includes the evaluation of patient semen and seminal vesicle volume is missing and should be considered [28]. Importantly, patients must be scrupulously studied before TPLA in order to eventually detect the presence of prostate cancer, which is incidentally diagnosed after standard BPE treatments in a non-negligible percentage of cases [29], and cannot be diagnosed during minimally invasive BPE procedures. In this context, a preoperative serum PSA dosage < 4 ng/mL and a negative DRE, or a previous negative prostate biopsy in case of clinical suspicion of prostate cancer are strongly recommended. 

The limitations of the present systematic review are mainly related to the small number of studies and patients included, and to the level of evidence of the studies, which demonstrated low or intermediate quality according to the Ottawa-Newcastle scoring system, causing a non-negligible risk of bias, which is not adequate to provide high-level evidence. Additionally, due to the non-comparative nature of the six included studies, only a pooled analysis was performed, which showed a high heterogeneity between studies. Higher level comparative studies with longer follow-up duration, or even possibly randomized clinical trials comparing TPLA with other standard or minimally invasive approaches, should be designed to test the real efficacy of TPLA of the prostate.

## 5. Conclusions

Transperineal laser ablation of the prostate is an innovative minimally invasive treatment option for BPE that showed interesting and promising results in pilot studies, such as improvement in urodynamic parameters, relief of obstructive symptoms, preservation of sexual function, and low rates of major complications. These observations suggest that TPLA should be compared in randomized clinical trials with other standard treatment options for BPE, in order to assess its efficacy and safety profile.

## Figures and Tables

**Table 1:** Characteristics of the six included studies that evaluated the efficacy of transperineal laser ablation (TPLA) for benign prostatic enlargement (BPE).

Study	Type of Study	Recruitment Period	Inclusion Criteria	Exclusion Criteria	Number of Patients
Pacella, C.M. et al. [7]. Prostate Cancer and Prostatic Disease, 2019	Retrospective Multi-institution	NR	Age > 50 IPSS ≥ 12 PV > 30 mL (TRUS) Qmax < 15 mL/s PVR < 400 ml	Urethral stricture, previous prostatic surgery, neurological disorders, previous diagnosis of PCa	160
De Rienzo, G. et al. [8], European Urology, 2021	Prospective Single-institution	September 2018–March 2019	Age 40–90 IPSS ≥ 12 PV ≤ 100 mL lack of efficacy, intolerance, or poor compliance to previous medical therapy	Previous surgical treatment for BPH, indwelling catheter or intermittent catheterization, bladder stones, detrusor acontractility or hypocontractility (BCI < 50), urethral strictures, neurogenic bladder dysfunctions, previous diagnosis of BCa of PCa	21
Cai, H.J. et al. [9], Acta Radiologica, 2021	Retrospective Single-institution	June 2018–January 2020	Age > 50 IPSS ≥ 12 PV ≥ 30 mL (US or MRI) Qmax ≤ 15 mL/s PVR 50–400 mL	Previous bladder neck, urethral or prostate surgery, PSA ≥ 4 ng/mL, previous diagnosis of PCa, urethral strictures, neurological disorders, hypersensitivity to US contrast media	20
Manenti, G. et al. [10], European Radiology Experimental, 2021	Prospective Single-institution	May 2018–February 2020	Age > 50 IPPS ≥ 12 PV > 30 mL lack of efficacy, intolerance, or poor compliance to previous medical therapy	Urethral stricture, previous prostatic surgery, clinical or imaging findings suspicious for malignancy confirmed by biopsy, neurological disorders, large median lobe, indwelling catheter, previous diagnosis of BCa or PCa	44
Frego, N. et al. [11], World Journal of Urology, 2021	Prospective Single-institution	July 2019–January 2020	Age ≥ 45 IPPS ≥ 8 PSA < 4 ng/mL, previous negative prostate biopsy, or negative DRE Qmax 15 mL/s PVR ≤ 150 mL PV 30–100 mL	Previous bladder neck, urethral or prostatic surgery, previous diagnosis of BCa or PCa, neurological disorders, gross hematuria, active UTI	22
Sessa, F. et al. [12], Urology Video Journal, 2022	Prospective Single-institution	April 2021–December 2021	NR	NR	30

Abbreviations: NR, not reported; IPPS, International Prostatic Symptoms Score; PV, prostate volume; Qmax, maximum urinary flow; PVR, postvoiding residue; PCa, prostate cancer; BPH, benign prostatic hyperplasia; BCI, bladder contractility index; BCa, bladder cancer; US, ultrasonography; MRI, magnetic resonance imaging; PSA, prostate-specific antigen; UTI, urinary tract infections.

**Table 2:** Technical aspects of transperineal laser ablation (TPLA) procedure for benign prostatic enlargement (BPE) in the six included studies.

Study	Number and Type of Needles	Needle Positioning	Number and Type of Fibers	Energy Released	Power
Pacella, C.M. et al. [7]. Prostate Cancer and Prostatic Disease, 2019	1 needle per lobe 1 more needle if PV > 40 mL 21 G Chiba	8–10 mm from the urethral wall 15 mm from the bottom of the bladder 10 mm from the prostatic capsule 8–10 mm between two needles if more in the same lobe	1 fiber each needle Bare optic quartz fiber caliber: 300 μm	1800 Joule/ fiber/firing	Continuous 3 Watt
De Rienzo, G. et al. [8], European Urology, 2021	1 needle per lobe 1 more needle if PV > 55–60 mL 21 G Chiba	8 mm from urethra lumen 8 mm from the prostatic capsule >15 mm from the bladder neck 10–15 mm between two needles if more in the same lobe	1 fiber each needle Flat-tipped optical fiber caliber: 300 μm	1800 Joule/ fiber/firing	Initial 4.5 Watt Final (after 1–2 min) 3.5 Watt
Cai, H.J. et al. [9], Acta Radiologica, 2021	1 needle per lobe 21 G Chiba	8–10 mm from urethra lumen >15 mm from the bladder neck 15–20 mm between two needles if more in the same lobe	1 fiber each needle caliber: 300 μm	1800 Joule/ fiber/firing	Continuous 3 Watt
Manenti, G. et al. [10], European Radiology Experimental, 2021	1 needle per lobe 1 more needle if PV ≥ 45 mL 21 G Chiba	10 mm from urethral lumen 15 mm from the bladder wall 10 mm from the prostatic capsule 8–10 mm between two needles if more in the same lobe	1 fiber each needle Bare optical quartz fiber caliber: 272 μm	1800 Joule/ fiber/firing in 400–600 s	Initial 5 Watt Final (after 2 min) 3 Watt
Frego, N. et al. [11], World Journal of Urology, 2021	1 needle per lobe 1 more needle if PV ≥ 60 mL 21 G Chiba	10 mm from urethral lumen 15 mm from the bladder neck 10 mm from the prostatic capsule 10–15 mm between two needles if more in the same lobe	1 fiber each needle Flexible quartz optical fiber caliber: 272 μm	1800 Joule/ fiber/firing in 600 s	Continuous 3 Watt
Sessa, F. et al. [12], Urology Video Journal, 2022	1 needle per lobe 1 more needle if PV > 80mL 21 G Chiba	8 mm from the urethra 15 mm from the bladder neck	1 fiber each needle Flat-tipped flexible optical quartz fiber caliber: 300 μm	1400 Joule/ fiber/firing	Initial 5 Watt Final (after 2 min) 3.5 Watt

**Table 3:** Summary of the main perioperative features of transperineal laser ablation (TPLA) for benign prostatic enlargement (BPE) in all the six included studies.

Study	Anesthesia	Antibiotic Prophylaxis	Operative Time	Ablation Time	Length of Hospital Stay	Previous Medical Therapy	Therapy at Discharge	Catheterization Time
Pacella, C.M. et al. [7]. Prostate Cancer and Prostatic Disease, 2019	Sedations (Midazolam 3 mg) + Local anesthesia (Lidocaine 20 mL/2%, perineal and periprostatic)	Ciprofloxacin 500 mg	44.0 (±12.9) min	NR	1.8 (±0.4) days	NR	NR	NR
De Rienzo, G. et al. [8], European Urology, 2021	Sedations + Local anesthesia (Lidocaine 20 mL/2%, perineal and periprostatic)	Oral cephalosporines or fluoroquinolones (1 h before and for 7 days after procedure)	36.0 (±9.5) min	NR	20.8 (±3.6) h	14 alpha-blockers (66.7%) 10 5-ARI (47.6%) 8 combination therapy (38.1%)	Antibiotic for 5 days; prednisone 25 mg for 15 days with subsequent tapering of the dose; bromelain for 30 days; Alpha-blockers for 30 days	8.7 (±2.5) days
Cai, H.J. et al. [9], Acta Radiologica, 2021	Local anesthesia (Lidocaine 20 mL/2%, perineal and periprostatic)	NR	60.9 (±10.8) min	42.6 (±9.9) min	1.5 (±0.5) days	NR	NR	16.5 (±4.2) days
Manenti, G. et al. [10], European Radiology Experimental, 2021	Local anesthesia (Lidocaine 20 mL/2%, perineal and periprostatic)	Levofloxacin 500 mg (1 h before and for 5 days after procedure)	28.2 (±10.6) min	NR	NR	NR	Antibiotic for 5 days; Acetaminophen 1000 mg if necessary; Prednisone 25 mg for 5 days with subsequent dose tapering; Alpha-blockers for 30 days	NR
Frego, N. et al. [11], World Journal of Urology, 2021	Sedations (Midazolam 4 mg) + Local anesthesia (Lidocaine 20 mL/2%, perineal and periprostatic)	Levofloxacin 500 mg (1 day before and for 5 days after procedure)	NA	1033 (600–1133) s	NR	22 alpha-blockers (100%) 6 combination therapy (27.3%)	Antibiotic for 5 days; Dexamethasone 8 mg and Ketoprofen 100 mg for 7 days	11.3 (±11.5) days
Sessa, F. et al. [12], Urology Video Journal, 2022	Sedations (benzodiazepine oral solution) + Local anesthesia (Lidocaine 20 mL/2% perineal and periprostatic) + Lidocaine/prilocaine 5% cream on perineum skin	Cephazolin 2 g (1 h before the procedure) and Cefixime 400 mg for 7 days after the procedure	31.5 (28–37) min	NR	6.4 (5.9–7.2) h	16 alpha-blockers (53.3%) 6 5-ARI (20.0%) 4 combination therapy (13.3%)	Antibiotic for 7 days; Gastroprotective therapy (pantoprazole 20 mg daily) for 7 days; Ibuprofen 600 mg twice a day for 7 days	7 (7–8) days

**Table 4:** Baseline patient characteristics and functional outcomes at 1, 3, 6, and 12 months of follow-up, for all the six included studies that evaluated the efficacy of transperineal laser ablation (TPLA) for benign prostatic enlargement (BPE).

Study	Time	N	AGE ^1^	BMI (Kg/m^2^) ^1^	PV (mL) ^1^	PSA (ng/mL) ^1^	Q_max_ (mL/min) ^1^	PVR ^1^	IPSS ^1^	IIEF-5 ^1^	MSHQ-EjD3 ^1^	QoL ^1^
Pacella, C.M. et al. [7]. Prostate Cancer and Prostatic Disease, 2019	Baseline	160	69.8 (±9.6)	NR	75.0 (±32.4)	NR	8.0 (±3.8)	89.5 (±84.6)	22.5 (±5.1)	NR	NR	4.5 (±1.1)
	1 month				NR	NR	NR	NR	NR	NR	NR	NR
	3 months				NR	NR	NR	NR	NR	NR	NR	NR
	6 months				27.2 (±14.9) *	NR	14.3 (±3.9) *	27.2 (±44.5) *	7.7 (±3.3) *	NR	NR	1.8 (±1) *
	12 months				58.8 (±22.9) *	NR	15.0 (±4.0) *	17.8 (±51.0) *	7.0 (±2.9) *	NR	NR	1.6 (±0.9) *
De Rienzo, G. et al. [8], European Urology, 2021	Baseline	21	62 (54–59)	27 (25–28)	40 (40–50)	2.0 (1.3–3.0)	9.2 (±3.4)	81.8 (±62.6)	18.3 (±3.9)	17.9 (±6.9)	5.7 (±4.5)	4.1 (±1.0)
	1 month					3.0 (±1.9)	12.1 (±6.4) *	37.4 (±25.7)	12.0 (±5.6) *	17.4 (±5.0)	9.6 (±4.1) *	2.4 (±1.6) *
	3 months				NR	1.7 (±0.8)	13.3 (±6.7) *	18.7 (±21.2) *	8.3 (±3.8) *	17.7 (±6.7)	6.8 (±3.5) *	1.4 (±0.9) *
	6 months				NR	1.7 (±0.8)	13.9 (±6.2) *	14.0 (±16.7) *	6.1 (±2.6) *	18.3 (±5.7)	8.6 (±3.1) *	1.7 (±0.8) *
	12 months				NR	NR	NR	NR	NR	NR	NR	NR
Cai, H.J. et al. [9], Acta Radiologica, 2021	Baseline	20	73.9 (±9.2)	NR	70.8 (±23.8)	NR	8.5 (±3.0)	78.7 (±58.8)	22.7 (±5.3)	NR	NR	4.9 (±1.7)
	1 month				NR	NR	NR	NR	NR	NR	NR	NR
	3 months				NR	NR	NR	NR	NR	NR	NR	NR
	6 months				54.7 (±20.9) *	NR	15.3 (±4.8) *	30.3 (±34.2) *	9.1 (±3.2) *	NR	NR	2.3 (1±.3) *
	12 months				NR	NR	NR	NR	NR	NR	NR	NR
Manenti, G. et al. [10], European Radiology Experimental, 2021	Baseline	44	72.1 (±6.1)	NR	102.4 (±36.3)	7.3 (±1.8)	7.6 (±4.2)	138.4 (±40.8)	18.5 (±5.5)	21 (±4)	4.9 (±3.7)	5.8 (±1.4)
	1 month				NR	NR	NR	NR	NR	NR	NR	NR
	3 months				NR	NR	NR	NR	NR	NR	NR	NR
	6 months				NR	NR	NR	NR	NR	NR	NR	NR
	12 months				48.1 (±19.2) *	2.1 (±0.8) *	16.2 (±4.9) *	18.8 (±8.5) *	6.2 (±3.8) *	22.0 (±3.2)	7.7 (±3.2) *	2.1 (±1.1) *
Frego, N. et al. [11], World Journal of Urology, 2021	Baseline	22	61.9 (55–65.5)	27.2 (24.8–28.6)	65 (46.5–81)	2.2 (1.4–4.5)	9 (5–12.5)	60 (25–107.5)	22 (19.5–25.3)	22 (16.5–24)	NR	4 (4–5)
	1 month				NR	NR	NR	NR	NR	NR	NR	NR
	3 months				46 (28.4–69)	NR	12 (9–16.5) *	39 (10–87.5)	8 (4.5–11) *	22 (19.5–24)	NR	1 (0.5–2) *
	6 months				42.3 (39.5–59) *	NR	15 (11.5–20.5) *	40 (16–63)	5 (3–8.5) *	23 (20.5–24)	NR	1 (0–2) *
	12 months				41.5 (36.3–55) *	NR	20.5 (14.3–27.8) *	30 (5–50)	6 (4.3–7) *	21.5 (17.3–23.8) *	NR	1 (1–2) *
Sessa, F. et al. [12], Urology Video Journal, 2022	Baseline	30	72 (64–79)	28 (24–31)	42 (40–53)	1.64 (0.56- 2.43)	9.5 (7.6–11.2)	100 (70–150)	21.5 (18–27.8)	16 (7.5–23.5)	5 (3–7.4)	4 (4–5)
	1 month					1.52 (0.93–1.87)	10.5 (8–16)	50 (20–100)	14.5 (12–17.8)	18 (15–24)	7.5 (4–13.1)	3 (2–3.75)
	3 months					1.51 (0.97–1.79)	14.2 (11.2–16.3)	40 (25–70)	13 (11.3–16.4)	23 (17.5–25)	8.9 (7–16.4)	2 (1.75–2.25)
	6 months					NR	NR	NR	NR	NR	NR	NR
	12 months					NR	NR	NR	NR	NR	NR	NR

Abbreviations: BMI, body mass index; PV, prostate volume; PSA, prostate-specific antigen; PVR, postvoiding residue; IPPS, International Prostatic Symptoms Score; IIEF, International Index of Erectile Functions; MSHQ-EJD, Male Sexual Health Questionnaire-Ejaculatory Dysfunction; QoL, quality of life. ^1^ Mean (SD) or median (IQR) * *p* < 0.05 (compared with baseline value).

## Data Availability

The full data set and code for statistical analyses are available upon request from the corresponding author.

## Supplementary Materials

The following supporting information can be downloaded at: https://www.mdpi.com/article/10.3390/jcm12051860/s1, Figure S1: Risk of bias summary showing review authors’ judgments about each risk of bias item for each included study; Figure S2: Forest plots illustrating the pooled mean and 95% confidence interval (CI) for postvoiding residue (PVR) and quality of life (QoL) at baseline and during follow-up.; Figure S3: Forest plots illustrating the pooled mean and 95% confidence interval (CI) for International Index of Erectile Function (IIEF-5) and Male Sexual Health Questionnaire-Ejaculatory Dysfunction (MSHQ-EjD) at baseline and during follow-up; Table S1: PICOTS (Population, Intervention, Comparators, Outcomes, Timing, and Setting) format; Table S2: Level of evidence for the evaluated studies according to GRADE and Oxford systems, and quality of the studies assessed with Newcastle-Ottawa scale; Table S3: Summary of rates and type of complications or sequelae and associated management in patients treated with transperineal laser ablation (TPLA) for benign prostatic enlargement (BPE) in the six included studies.
