# Supplementary material for: Transperineal Laser Ablation for Benign Prostatic Enlargement: A Systematic Review and Pooled Analysis of Pilot Studies
Source: J Clin Med. 2023 Feb 26;12(5):1860. doi: 10.3390/jcm12051860 (PMC10003190; doi:10.3390/jcm12051860)
Supplement: Supplementary file 1 [file jcm-12-01860-s001.zip › jcm-2201599-supplementary.pdf]

Supplementary Materials

Table S1. PICOTS (Population, Intervention, Comparators, Outcomes, Timing, and Setting) format.

**Population**	Age: >40 yrs.<br>Diagnoses: moderate/severe Lower Urinary Tract Symptoms (LUTS) due to Benign Prostate Enlargement (BPE) and lack of efficacy, intolerance, or poor compliance to previous medical therapies.
**Interventions**	Transperineal Laser Ablation (TPLA) for Benign Prostatic Enlargement
**Comparators**	None
**Outcomes**	Improvement in urodynamic parameters (maximum urinary flow (Q_{max}) and postvoiding residue (PVR))<br>Symptom relief (IPPS, International Prostatic Symptoms Score questionnaire)<br>Preservation of ejaculatory function (MSHQ-EjD, Male Sexual Health Questionnaire-Ejaculatory Dysfunction)<br>Complication rates
**Type of studies**	Prospective or retrospective studies including more than 20 patients
**Timing and setting**	Any timepoint and setting

Table S2. Level of evidence for the evaluated studies according to GRADE and Oxford systems, and quality of the studies assessed with Newcastle-Ottawa scale.

**Study ID**	**Year**	**Level of Evidence According to:**		**Quality**
		**Grade System**	**Oxford System**	
Pacella et al.,<br>Prostate Cancer and Prostatic Disease	2019	2	2b	5
De Rienzo et al.,<br>European Urology	2021	2	2b	6
Cai et al.,<br>Acta Radiologica	2021	2	2b	6
Manenti et al.,<br>European Radiology Experimental	2021	2	2b	6
Frego et al.,<br>World Journal of Urology	2021	2	2b	5
Sessa et al.,<br>Urology Video Journal	2022	2	2b	5

Table S3. Summary of rates and type of complications or sequelae and associated management in patients treated with transperineal laser ablation (TPLA) for benign prostatic enlargement (BPE) in the six included studies.

**Study**	**N**	**Type and Number of Complications**	**Management**	**Complication Rate**	**Clavien–Dindo**
Pacella et al.,<br>Prostate Cancer and Prostatic Disease,<br>2019	160	3—transient hematuria<br>3—acute urinary retention<br>1—orchitis<br>1—prostatic abscess	None<br>Bladder catheter for 15 days<br>Antibiotic<br>Antibiotic and percutaneous drainage	1.9%<br>1.9%<br>0.6%<br>0.6%	I<br>I<br>II<br>IIIa
De Rienzo et al.,<br>European Urology,<br>2021	21	1—prostatic abscess	Antibiotic and percutaneous drainage	4.8%	IIIa
Cai et al.,<br>Acta Radiologica,<br>2021	20	1—Intraoperative urethral burn	Bladder catheter for 25 days	5.0%	I
Manenti et al.,<br>European Radiology Experimental,<br>2021	44	1—prolonged hematuria<br>1—orchitis (ATB)<br>1—prostatic abscess	Bladder catheter for 7 days<br>Antibiotic<br>Antibiotic and percutaneous drainage	2.2%<br>2.2%<br>2.2%	I<br>II<br>IIIa
Frego et al.,<br>World Journal of Urology,<br>2021	22	8—dysuria spontaneously resolved<br>3—acute urinary retention<br>2—urinary tract infection	None<br>Bladder catheter for 7 days<br>Hospitalization and antibiotic	36.4%<br>13.6%<br>9.0%	I<br>I<br>II
Sessa et al.,<br>Urology Video Journal,<br>2022	30	NR	NR	NR	NR

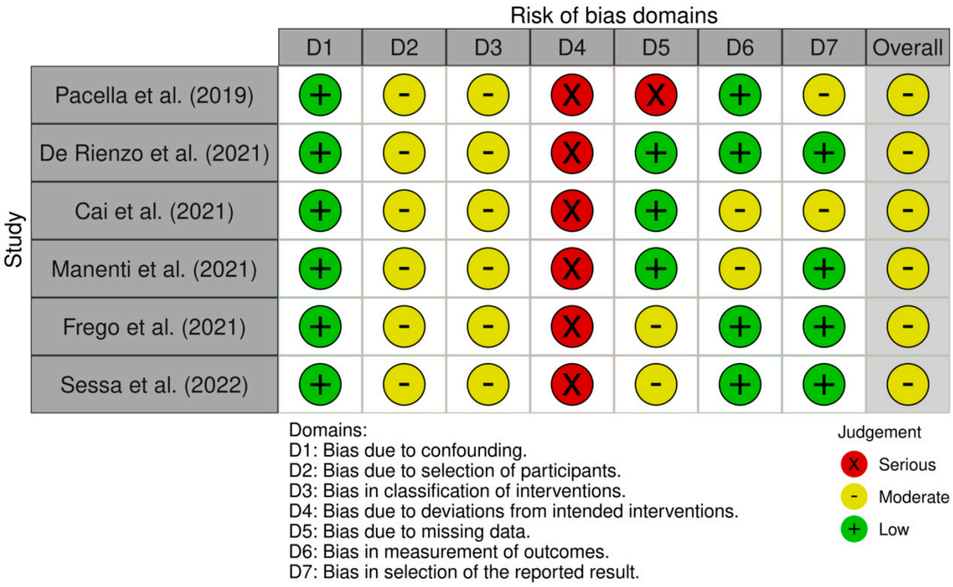

Figure S1. Risk of bias summary showing review authors' judgments about each risk of bias item for each included study.

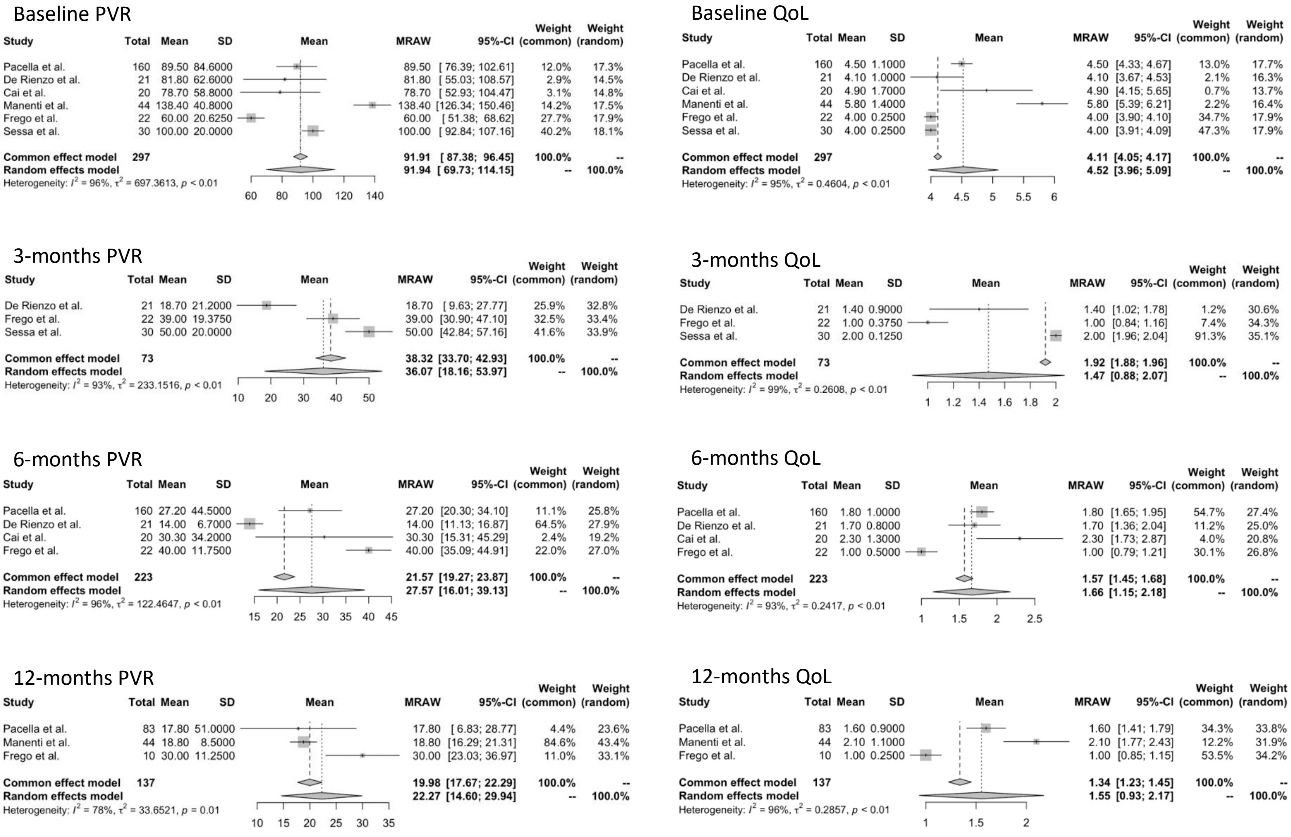

Figure S2. Forest plots illustrating the pooled mean and 95% confidence interval (CI) for postvoiding residue (PVR) and quality of life (QoL) at baseline and during follow-up.

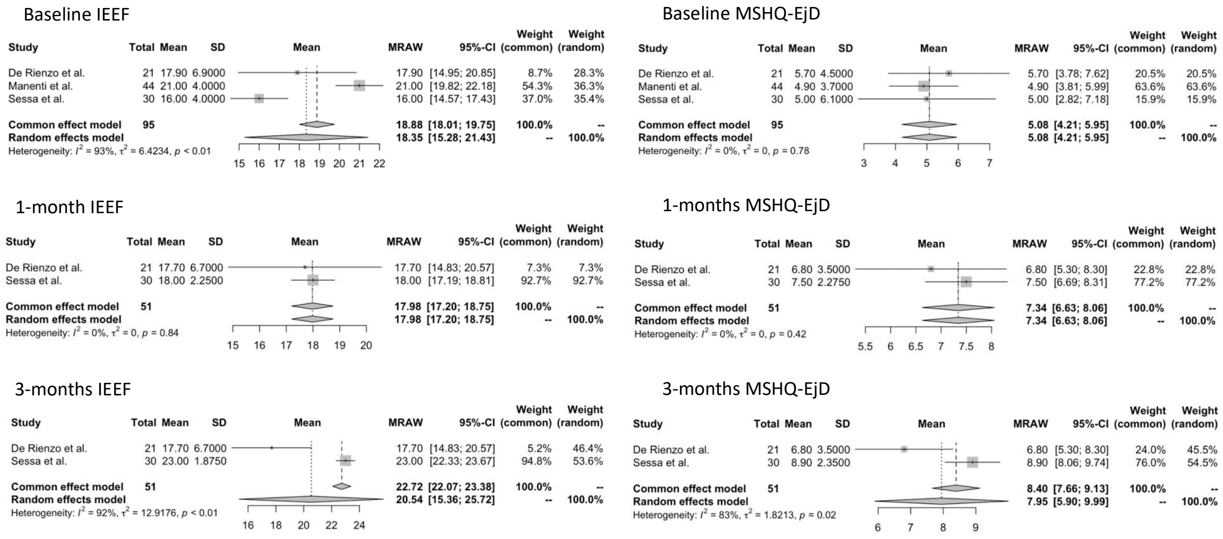

Figure S3. Forest plots illustrating the pooled mean and 95% confidence interval (CI) for International Index of Erectile Function (IIEF-5) and Male Sexual Health Questionnaire-Ejaculatory Dysfunction (MSHQ-EjD) at baseline and during follow-up.
